# Hemoptysis - a Rare Complication of Pacemaker Implantation

**Published:** 2008-02-01

**Authors:** Alexander Goldberg, Inna Rosenfeld, Alon Marmor

**Affiliations:** Sieff Government Hospital, Safed, Israel

**Keywords:** Hemoptysis, Instrumentation, Pacing

## Abstract

We describe a case of hemoptysis as a rare complication of pacemaker lead insertion via the axillary approach in a patient with difficult chest anatomy.

## Introduction

Implantation of pacemaker lead via subclavian puncture carries a small but significant risk of access-related complications that include pneumothorax, hemopneumothorax, lung laceration, inadvertent arterial puncture, air embolism, arteriovenous fistula, thoracic duct injury and brachial plexus injury. In order to minimize the risks of complications related to subclavian puncture the axillary access for lead insertion has been developed [1]. Here we describe a rare case of prolonged hemoptysis as a complication of transvenous lead placement using the axillary approach.

## Case report

An 86-year-old patient with recurrent syncope and sick sinus syndrome was referred to our institution for implantation of a permanent pacemaker. His past medical history was remarkable for the resection of his right lung done 45 years ago because of pulmonary tuberculosis. The patient was very slender; his chest was asymmetric. The chest X-ray was remarkable for hyperinflated left lung, the heart and great vessels were shifted rightward. Given the increased risk of pneumothorax, the left cephalic vein was our first choice for lead insertion; however it was too small to accommodate the lead. Therefore, the axillary approach was chosen. The attempt to puncture the left axillary vein with 18-gauge needle was performed under fluoroscopic guidance according to the described technique [1];  however, after the first needle pass the patient had 30-50 ml of hemoptysis. Both lung fields were carefully examined fluoroscopically, but no signs of pneumothorax were identified. The patient was observed for 15 minutes for respiratory distress or hemodynamic instability and, as neither was present, we decided to proceed with the planned procedure. With the second attempt, done with the needle directed more superficially, the left axillary vein was successfully canulated, the pacemaker lead was inserted via this vessel and positioned in the right ventricular apex. During the operation the patient occasionally had a small amount of hemoptysis, but he had no dyspnea or chest pain, his oxygen saturation, heart rate and blood pressure were stable. The chest X-ray performed upon completion of the operation showed no signs of pneumothorax or hemopneumothorax. The patient continued to have occasional hemoptysis during the next three days that eventually stopped spontaneously. The tuberculosis work-up was negative; as the patient was stable and the amount of hemoptysis was small, no additional imaging was done. The patient was discharged home on the fourth post-operative day in stable condition. The follow up visits at two and six weeks post implantation were unremarkable.

## Discussion

Hemoptysis is an exceedingly rare acute complication of pacemaker implantation. To the best of our knowledge only one case of hemoptysis following subclavian puncture for pacemaker lead insertion have been previously described [2]. The authors' assumption was that the inadvertent lung puncture has caused the brief episode of self-limited hemoptysis.

The axillary approach for transvenous pacemaker lead insertion is a safe and feasible alternative to more commonly used cephalic and subclavian approaches. Using this approach, the puncture is performed three fingerbreadths below the coracoid process and the axillary vein is usually entered at the medial border of the pectoralis minor muscle [1]. The axillary vein is extrathoracic, therefore no possibility for pneumothorax or hemopneumothorax exists. Also, the long-term consequences of sublavian crush syndrome are prevented with this approach. However, in cases of altered chest anatomy this approach may be difficult and not free of complications. In the case presented, both the hyperinflated left lung and very thin chest wall might have predispose the needle to go too deep and penetrate the lung tissue. Although the actual mechanism of hemoptysis in our case has not been established, we speculate that the needle created a fistulous tract between a small blood vessel and a bronchus. This tract could be a route for prolonged, although hemodinamically insignificant, hemoptysis. It is possible that using contrast venography to facilitate the axillary puncture could have been helpful in avoiding this rare complication.

## Conclusion

Hemoptysis is a rare but real access-related complication of pacemaker implantation. The implanting physician should not hesitate to implement contrast venography in cases with difficult anatomy to facilitate the venous puncture.
